# Sarcoidosis vs. Colon Cancer Metastasis: Diagnostic Dilemma and the Role of PET Scan in Monitoring Disease Activity

**DOI:** 10.1155/2021/5529523

**Published:** 2021-04-20

**Authors:** Raai Mahmood, Kadhim Al Banaa, Israa Ibrahim, Ahmed Hashim, Luis Torregrosa

**Affiliations:** ^1^Graduate Program, Ohio State University, Columbus, OH, USA; ^2^Hemostasis and Thrombosis Department, University of California, San Diego, CA, USA; ^3^Faculty of Medicine, Luthran Hospital, Fort Wayne, IN, USA; ^4^Department of Internal Medicine, Beaumont Hospital, Dearborn, MI, USA; ^5^Department of Rheumatology, Beaumont Hospital, Dearborn, MI, USA

## Abstract

Sarcoidosis is a systemic inflammatory disorder characterized by “noncaseating granulomas.” It primarily affects the lungs, but multiple other organs can be involved. Sarcoidosis has been increasingly reported in association with cancer. It can precede, follow or occur at the same time as the diagnosis of cancer. We report a case of sarcoidosis that was diagnosed concomitantly with colon cancer, highlighting the diagnostic dilemma of sarcoidosis vs. cancer metastasis, the relationship between the two, and the value of PET scan in follow-up and monitoring of disease activity.

## 1. Introduction

Sarcoidosis comes from the Greek word “Sarco,” which means flesh [1]. It is a systemic inflammatory disorder characterized by “noncaseating granulomas.” It primarily affects the lungs, but any other organ can be involved.

In the last few decades, sarcoidosis has been increasingly reported in association with cancer. It can precede or occur at the same time of cancer diagnosis. Sarcoidosis was also reported several years after cancer diagnosis and/or flourished after initiation of chemotherapy or radiotherapy.

Follow-up of sarcoidosis is complicated and depends largely on the organ involved. Symptoms, signs, and inflammatory markers are the corner stones of follow-up; however, PET scan, being a reliable method of measuring disease activity and distinguishing active granuloma from fibrosis, proves to be a useful tool.

## 2. Case Presentation

The patient was a previously healthy 49-year-old Caucasian female who presented to the hospital with abdominal pain associated with nausea, vomiting, and weight loss. CT of her abdomen showed circumferential wall thickening of the ascending colon, highly suspicious for colonic carcinoma with associated pericolic and upper abdominal lymphadenopathy and splenomegaly with several splenic hypodensities suggestive of metastases was also noted.

The patient underwent laparotomy with hemicolectomy, histopathology revealed ascending colon adenocarcinoma. Five lymph nodes were positive for metastatic carcinoma and multiple sarcoidal noncaseating granulomas were also noted.

PET (positron emission tomography) scan following surgery revealed mediastinal bilateral hilar lymphadenopathy and splenomegaly with marked abnormally increased metabolic activity suspicious for metastasis. Prior to her presentation, there was no chest imaging available for comparison.

Splenic biopsy showed multiple granulomas with focal necrosis. Stain was negative for fungal and acid-fast microorganisms.

Blood work at the time showed Ca of 9.1 mg/dl, Cr of 0.9 mg/dl, CEA level of 9.6 ng/ml, and ACE level of 45 nmol/ml/min. CRP and ESR were not checked as they could be falsely elevated in postoperative state. Given the absence of respiratory symptoms and minimally elevated serum ACE level, no treatment was deemed necessary for asymptomatic incidental sarcoidosis and the patient was started on chemotherapy to treat cancer.

**Figure 1:**
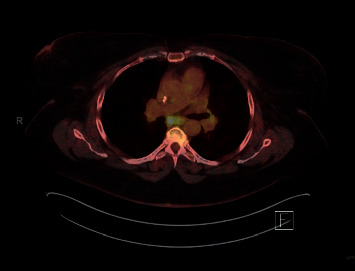
2/2017 hypermetabolic activity within the subcarinal lymph nodes.

**Figure 2:**
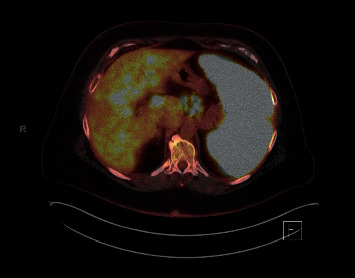
2/2017 significant abnormal radiotracer uptake within the spleen and scattered foci of hypermetabolic activity within the liver.

**Figure 3:**
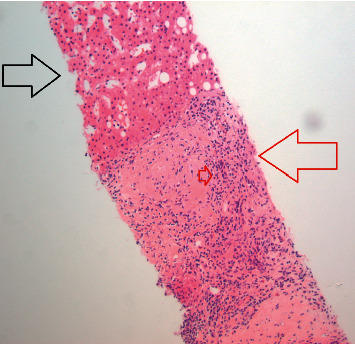
Low-power view showing benign liver tissue (black arrow) and granulomas (red arrow). The small red arrow shows palisading histiocytes surrounding a central fibrous area of the granuloma.

Two years later, a follow-up PET scan revealed increased lymphadenopathy in multiple areas including the left axilla and the left mammographic chain and mesenteric lymphadenopathy. Lesions were also noted in the liver and spinous processes of L3-L4. Progression of her splenic involvement was also noted (see Figures 1 and 2 ). Liver biopsy at the time showed only noncaseating granulomas with no evidence of malignancy (see Figure 3).

At that time, her CEA level had increased from 3 to 14.2 ng/ml and ACE level was >120 nmol/ml/min, ESR was>100 mm/hr, CRP was 15.5 mg/L, and Cr was 1.7 mg/dl. QuantiFERON-TB Gold and ANCA antibodies were all negative.

Given the presence of biopsy-proven sarcoidosis with elevated ACE level, compatible imaging, and the fact that serum CEA can also be elevated sarcoidosis [2], the patient was started on 40 mg of prednisone for treatment of sarcoidosis flare. Following two months of therapy, blood work showed significant improvement. Serum ACE level was down to normal at 35 nmol/ml/min, Cr improved to 1.01 mg/dl, and CRP decreased to 12.4 mg/L; however, CEA increased to 98 ng/ml.

**Figure 4:**
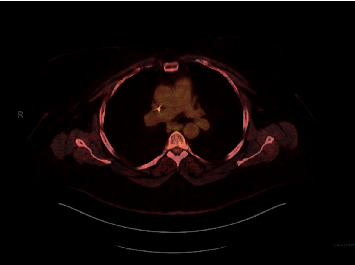
5/2017 interval near resolution of the bilateral hilar and subcarinal lymphadenopathy.

**Figure 5:**
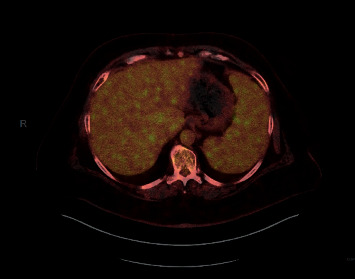
5/2017 the previously described diffuse scattered foci of hypermetabolic activity within the liver have significantly improved. The previously described abnormal activity within the spleen is not visualized anymore.

Repeated PET scan showed resolution of the hilar lymphadenopathy with improvement of the left axillary and liver metabolic activity. Previously reported increased splenic and bone metabolic activity had also resolved (see Figures 4 and 5). Mesenteric lymphadenopathy, however, had progressed. Biopsy of the mesenteric lymph node showed recurrence of cancer. The patient was referred to oncology to continue treatment for colon cancer, and, finally, hospice was offered given the poor response.

## 3. Discussion

Sarcoidosis is a multisystemic disorder of unknown cause characterized by the presence of granulomas in different organs. Ninety-five percent of patients will present with intrathoracic involvement, and fifty percent will have extrathoracic involvement, most commonly involving the skin and eyes [1]. Gastrointestinal involvement is uncommon and estimated to be less than 1% with stomach being the most common site reported [3]. Other sites involved include the liver, spleen, peritoneum, and lymph nodes.

Diagnosis of sarcoidosis is established when compelling clinical and radiological features are present in combination with histopathological evidence of noncaseating granulomas provided that other causes of granuloma including mycobacterial and fungal infections are excluded [1].

Imaging plays an essential part in the diagnosis of sarcoidosis and in assessing disease severity. Chest X-ray can help the diagnosis and staging of pulmonary sarcoidosis. CT can help detect lesions in multiple parts of the body; however, newer imaging modalities like PET CT have the advantage of detecting active inflammation by measuring increased glucose metabolism in those areas. Decreased tracer uptake on subsequent PET scans in response to treatment proves PET scans to be an important tool to monitor disease activity. [4].

The association between sarcoidosis and malignancy is a controversial topic. Sarcoidosis can precede, follow, or occur at the same time of diagnosis of cancer. A study of Danish cohorts who were followed for over thirty years showed an increased risk of cancer both short term and long term after diagnosis [5]. A more recent systematic review published in 2015 also showed a significant association between sarcoidosis and cancer with an increased risk for skin, hematological, and GI malignancy [6]. Relative risk for colorectal cancer associated with sarcoidosis was reported to be 1.33 [6].

The relationship between sarcoidosis and cancer takes many forms [7]: the sarcoidosis lymphoma syndrome in which lymphoma or other hematological malignancies are diagnosed within one to two years of sarcoidosis diagnosis. Other forms include the appearance of sarcoidosis following solid cancer diagnosis or sarcoidosis flare that occurs as a potential side effect of cancer therapy such as cisplatin [8] and interleukin [9].

It can occur as paraneoplastic syndrome related to cancer and is defined as sarcoidosis that is coincidental or diagnosed within one year of cancer diagnosis.

Pathophysiological interpretation implied a potential role of cytokines and angiogenesis [10]. Other theories suggested a state of anergy (poor response to antigens) known as the “immune paradox” of sarcoidosis, which results in diminished cellular immunity [11, 12].

In an attempt to characterize cancer cases associated with sarcoidosis, Spiekermann et al. identified 59 cases of solid tumors and sarcoidosis reported in the literature up to 2017, of which 24 cases of sarcoidosis diagnosed at the same time with malignancy [13]. Of the 59 cases, seven cases had GI malignancy.

In our case, sarcoidosis was diagnosed at the same time with colon cancer. Colonic lymph nodes were the first site at which sarcoidosis was discovered. It is unclear if sarcoidosis had preceded or occurred in response to colon cancer given the lack of symptoms, signs, and imaging prior to cancer diagnosis; nevertheless, the fact that her sarcoidosis was aggravated at the same time of recurrence of malignancy implies a potential role of cancer or chemotherapy in modulating sarcoidosis activity.

In this case, the patient initially had mildly elevated serum ACE level with no symptoms and no signs of end organ damage, so priority was given to cancer treatment and no therapy was deemed necessary to treat sarcoidosis. However, she returned two years later with significant elevation of serum ACE level combined with evidence of kidney injury, elevated biomarkers, imaging evidence of increased metabolic activity involving the chest, abdomen, bone, liver, and spleen, and biopsy-proven sarcoidosis, almost all of which had improved or completely resolved after initiation of immune suppressive therapy except for the abdominal lymph nodes that were proved to be cancerous later on.

Differentiating cancer recurrence from sarcoidosis was extremely challenging in this case, and tissue biopsy is almost always needed to rule out cancer recurrence. Follow-up of sarcoidosis can also be challenging, and testing depends largely on the organs involved. Serum ACE level is elevated in about 90% of patients and is known to be a marker of disease activity anticipated to normalize with therapy [1]. Yet, serum ACE level is nonspecific and can also be elevated in other rheumatologic diseases as well as in some infections and in lung cancer. Serum ACE level cannot solely determine the presence of sarcoidosis flare and does not always correlate with increased metabolic activity on PET scan [14], which, on the other hand, can help assess disease activity and serves as a valuable tool for follow-up but is also nonspecific. PET scan, however, can differentiate active granuloma from fibrosis which is of importance in determining appropriate therapy [4].

This case represents a diagnostic dilemma of cancer metastasis vs. sarcoidosis and the role of PET scan to monitor disease activity and response to treatment; it also imposes a question of a potential two-way relationship between cancer and sarcoidosis.

Should a patient with sudden sarcoidosis flare be screened for occult malignancy? Do patients with established sarcoidosis need to be monitored more closely after cancer diagnosis or treatment in anticipation of disease flare? The answer needs further investigation.

## Data Availability

Data used to support this case report are available from the corresponding author upon request.
